# Correction: Adverse birth outcome and associated factors among diabetic pregnant women in Ethiopia: Systematic review and meta-analysis

**DOI:** 10.1371/journal.pone.0249652

**Published:** 2021-03-30

**Authors:** Demeke Mesfin Belay, Wubet Alebachew Bayih, Abebaw Yeshambel Alemu, Aklilu Endalamaw Sinshaw, Demewoz Kefale Mekonen, Amare Simegn Ayele, Wasihun Hailemichael Belayneh, Henok Andualem, Biniam Minuye Birihane

The eighth author’s name appears incorrectly. The correct name is: Henok Andualem.

The ORCID iD is missing for the eighth author. Please see the author’s ORCID iD here:

- Author Henok Andualem’s ORCID iD is: 0000-0001-6883-9548 (https://orcid.org/0000-0001-6883-9548).

## References

[1] Belay DM, Bayih WA, Alemu AY, Sinshaw AE, Mekonen DK, Ayele AS, et al. (2020) Adverse birth outcome and associated factors among diabetic pregnant women in Ethiopia: Systematic review and meta-analysis. PLoS ONE 15(11): e0241811. 10.1371/journal.pone.0241811 33170888 PMC7654793

